# Dose Schedule Optimization and the Pharmacokinetic Driver of Neutropenia

**DOI:** 10.1371/journal.pone.0109892

**Published:** 2014-10-31

**Authors:** Mayankbhai Patel, Santhosh Palani, Arijit Chakravarty, Johnny Yang, Wen Chyi Shyu, Jerome T. Mettetal

**Affiliations:** Drug Metabolism and Pharmacokinetics, Takeda Pharmaceuticals International Co., Cambridge, Massachusetts, United States of America; Texas Tech Univ School of Pharmacy, United States of America

## Abstract

Toxicity often limits the utility of oncology drugs, and optimization of dose schedule represents one option for mitigation of this toxicity. Here we explore the schedule-dependency of neutropenia, a common dose-limiting toxicity. To this end, we analyze previously published mathematical models of neutropenia to identify a pharmacokinetic (PK) predictor of the neutrophil nadir, and confirm this PK predictor in an *in vivo* experimental system. Specifically, we find total AUC and C_max_ are poor predictors of the neutrophil nadir, while a PK measure based on the moving average of the drug concentration correlates highly with neutropenia. Further, we confirm this PK parameter for its ability to predict neutropenia *in vivo* following treatment with different doses and schedules. This work represents an attempt at mechanistically deriving a fundamental understanding of the underlying pharmacokinetic drivers of neutropenia, and provides insights that can be leveraged in a translational setting during schedule selection.

## Introduction

Balancing antitumor efficacy with toxicity remains a fundamental challenge in the development of antineoplastic agents, both for traditional chemotherapeutics and for the newer generation of targeted therapies. Therefore, a better understanding of the relationship between schedule and toxicity assumes a critical role in development of novel agents; as such an understanding would permit the rational selection of dosing schedules during the early clinical development of oncology drugs that could decrease the likelihood of adverse events while maintaining drug exposure and efficacy.

A common side-effect for many oncology drugs, (including both chemotherapeutic as well as targeted therapies), is hematological toxicity or myelosuppression [1]–[5]. In many cases, this hematological toxicity is clearly mechanism-related, as bone marrow stem cells represent a rapidly dividing population of cells that is vulnerable to antineoplastic agents that interfere with cell division and survival. Of the hematological toxicities, neutropenia is one of the most common reasons for chemotherapy delay and dose reduction [6], [7]. A significant drop in absolute neutrophil counts (ANC) has been demonstrated to be linked to the incidence of serious adverse events (such as sepsis, febrile neutropenia and other life-threatening infections). While neutropenia is serious, it is also easily monitored and anticipated. There is often a strong schedule-dependent component to neutropenia, for example with taxanes in breast cancer, where weekly administration of drug resulted in a reduced toxicity profile relative to once-every-three weeks administration [8]–[12]. The active design of dosing schedules to limit the incidence of neutropenia, therefore, holds the potential of significant impact in the development of novel antineoplastic agents.

Much research has been focused on understanding the underlying PK driver of neutropenia in the clinic. A variety of model forms have been used to describe the relationship between neutropenia and overall drug exposure, such as linear [13]–[15], log-linear [16], nonlinear [17]–[19], and logistic regression [20]–[24] models. However, capturing the underlying relationship between pharmacokinetics (PK) and toxicity can often be challenging, as patient data is often analyzed from a single dose schedule and exposure metrics derived from these studies, such as AUC (Area Under the Curve) or C_max_ (maximum concentration of drug during the treatment period), are typically highly correlated with one another on any fixed schedule. This complexity often makes it complicated to extract a single simple PK parameter directly from empirical models of induction of neutropenia that have been described in the clinic [9], [11], [12], [25].

Here we take an alternative approach to mitigate the issue of correlations between PK parameters on experimentally tested schedules by utilizing prior knowledge built into semi-mechanistic models. These models have been successfully applied to predict not only the probability of occurrence, but also the time-course of neutropenia. These models work by combining a model of drug PK and compound specific myelosuppression potential with a model of the underlying biology of the blood-cell life cycle [26]–[34]. The semi-mechanistic models represent an advance over exposure metrics, as they provide a more complete description of ANC kinetics, and permit the comparison of different schedules for their potential to induce neutropenia. Interestingly, it is has been shown that models for chemotherapy-induced neutropenia can be based primarily on systemic (patient-specific) parameters for the bone marrow hematopoietic cascade, with a limited number of drug-specific parameters [26].

In this work we seek to identify an underlying PK parameter that predicts the degree of neutropenia by analyzing the dynamic and severity of neutropenia derived from the semi-mechanistic models of neutropenia in response to a variety of dosing schedules [26], [35]–[39]. First, we sought to determine whether there were any schedules that were less likely to induce neutropenia. Next, we analyzed the schedules to determine which PK parameter was most closely linked to the severity of neutropenia nadir. Finally, we tested the identified PK parameter on a set of *in vivo* neutrophil counts to demonstrate the validity and utility of the approach, independent of the model. The approach presented here is a natural extension of the foundational work done on semi-mechanistic models of neutropenia, and represents a mechanistic insight into the behavior of these models with practical implications for clinical study design and interpretation.

## Results

### Effect of Dosing Schedules on Docetaxel Induced ANC-nadir

**Figure 1:**
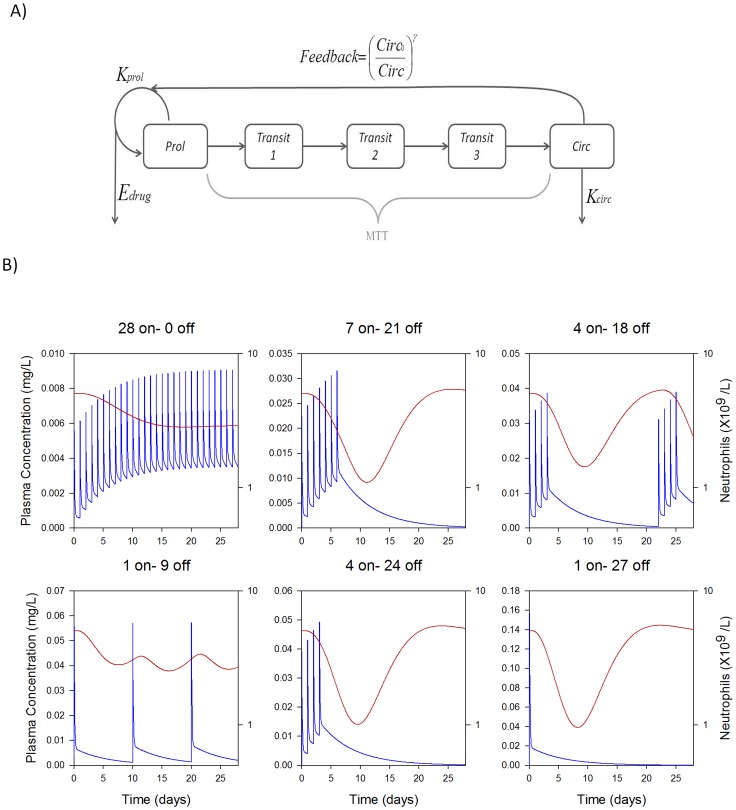
Model structure and simulated ANC time-course. (A) The structure of semi-mechanistic pharmacokinetic-pharmacodynamic Friberg model (from ref [26]) used to describe cytotoxic effect of drugs on proliferating neutrophils. Drug PK was linked to the semi-mechanistic PD model for neutrophil kinetics and unique PK-ANC profile for given drug at various schedules was generated. (B) Docetaxel PK-ANC simulation was carried out at an equivalent total dose to 100 mg/m^2^ every 21 days. The plots show the plasma PK profile (blue lines) and ANC upon treatment (red lines) for schedules of 28 on-0 off, 7 on-21 off, 4 on-18 off, 1 on-9 off, 4 on–24 off and 1 on-27off.

Clinical observations have shown that even for constant total dose per cycle, different dose schedules (e.g. frequency of dosing or infusion time) can lead to very distinct neutrophil dynamics and incidence of neutropenia [22], [27], [34], [36], [39]–[42]. Therefore, we used the Friberg model of neutropenia to study in detail how drug plasma time course correlated with incidence or severity by employing previously published semi-mechanistic population PK-PD models (Figure 1a) built from clinical ANC profiles following treatment with docetaxel [26], [34].

First, a range of different schedules were generated over an interval of eighty-four days keeping the total dose of docetaxel fixed (to match total exposure). The schedules tested were of the form of days-on/days-off, where an interval of continuous dosing (days-on) is followed by a holiday (days-off) before repeating the cycle on a fixed period. For example Q3W (once every three weeks) dosing can be expressed as one day of dosing within a 21 day period. Figure 1b illustrates several examples of the PK and ANC dynamics that result from various dosing schedules for a fixed total dose (equivalent to 100 mg/m^2^ given every three weeks).

**Figure 2:**
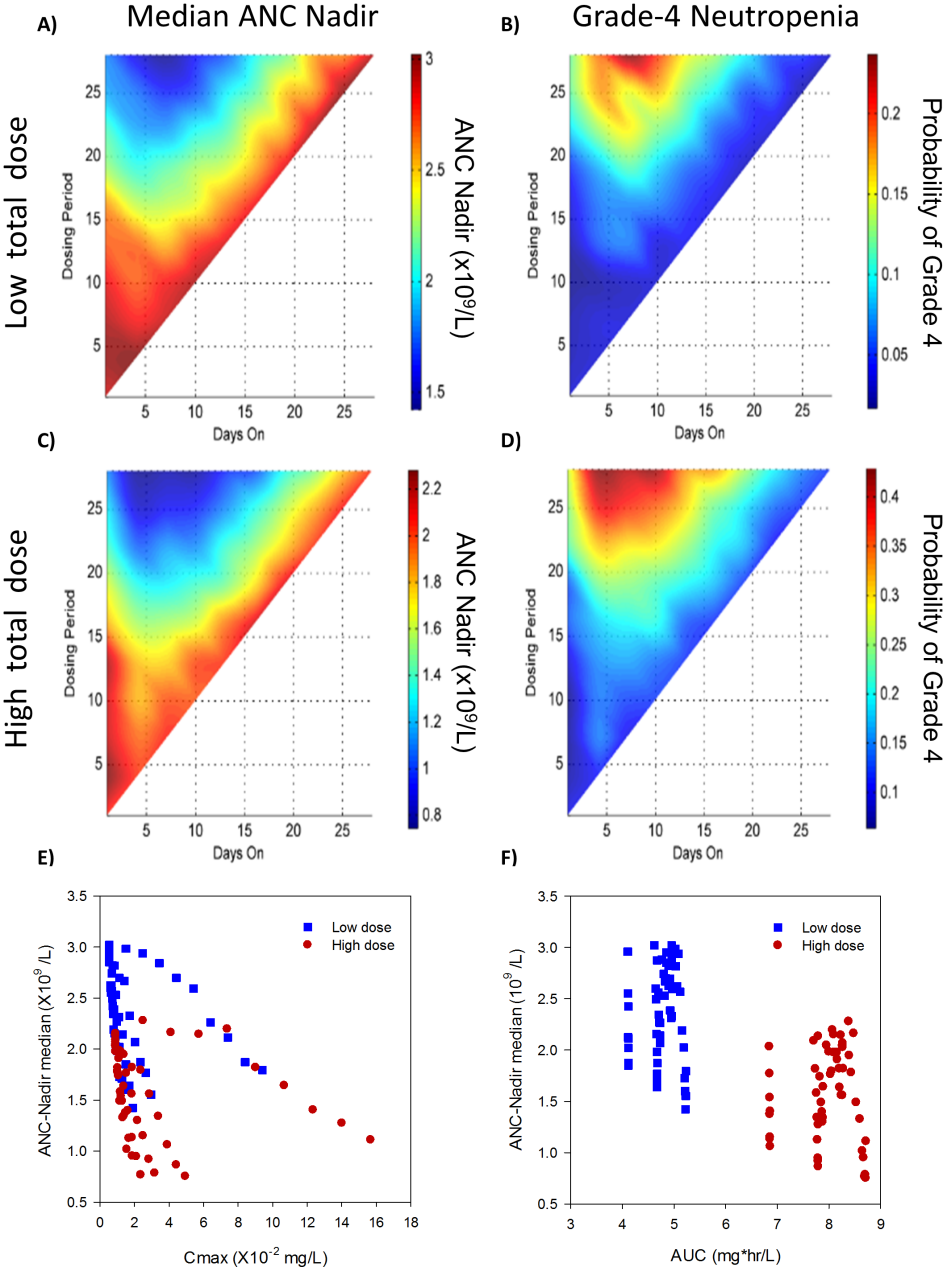
Effect of schedule on docetaxel induced neutropenia. Summary of population simulation of PK-ANC model for 1000 virtual individuals at two total dose levels of docetaxel shows constant dosing induces a less severe nadir than punctuated dosing. Population median ANC nadir for low (A) total dose and high (C) total dose as well as probability of grade 4 neutropenia (nadir <0.5×109/L) are shown for low (B) and high (D) dosing total dose levels. Each plotted captures schedules with the “days-on/days-off” format with number of consecutive days on in given treatment period shown on the X-axis and treatment period (sum of days on and days off) on the Y axis. Population estimation of median ANC nadir and probability of grade-4 neutropenia compared with common PK parameters across variety of schedules for low (blue) and high (red) total dose. (E) C_max_ plotted against median ANC nadir for each of the schedules tested and shows weak correlation of −0.32 (R^2^ = 0.10, *p*<0.01). (F) Total cycle AUC over all schedules shows overall correlation of −0.64 (R^2^ = 0.41, *p*<0.01) with median ANC nadir, but the correlation is mainly driven by the differences in total dose as it loses ability to predict neutropenia at a fixed total dose level (R^2^<0.01 and *p*>0.75 at low and high total dose).

ANC profiles were then simulated for a virtual population of 1000 patients for these schedules. Two different total dose levels, representing a high and low clinical dose range, were tested (Table 1). The results are shown in Figure 2a and 2c as a heat map of median ANC nadir (minimal absolute neutrophil count) for the simulated patient population under docetaxel treatment on a variety of schedules. Points along the diagonal in the plot (y = x) represent schedules with constant (QD) dosing. As can be seen in Figures 2a and 2c, the points in the upper left corner (e.g. a larger and less frequent dose) have a more severe ANC nadir compared to frequent dosing, and this trend is conserved both for low (Figure 2a) and high (Figure 2c) total dose.

**Table 1:** Equivalent total dose used in generation of simulated dosing schedules.

Dose Strength	Docetaxel	Topotecan	Etoposide
Low Dose	60 mg/m2 every 3 week	1.5 mg/m2 given as 30 min infusion on day 1,3 and 5 repeated 3 week	50 mg/m2 given as 30 min infusion for 5 days every 3 week
High Dose	100 mg/m2 every three week	4 mg/m2 given as 30 min infusion on day 1,3 and 5 repeated 3 week	100 mg/m2 given as 30 min infusion for 5 days every 3 week

The probability of an individual patient developing grade-4 neutropenia (defined as neutrophil count below 0.5×10^9^/L) is shown in Figure 2b and 2d with similar trends as neutropenia nadir. Median ANC nadir is a good predictor of incidence of grade-4 neutropenia within the virtual population with an R^2^ = 0.91 (*p*<0.0001). Since both the ANC nadir and the probability of developing grade-4 neutropenia show similar schedule-dependent trends, we next asked what correlation existed between the two measures across schedules.

### PK driver of neutropenia

**Figure 3:**
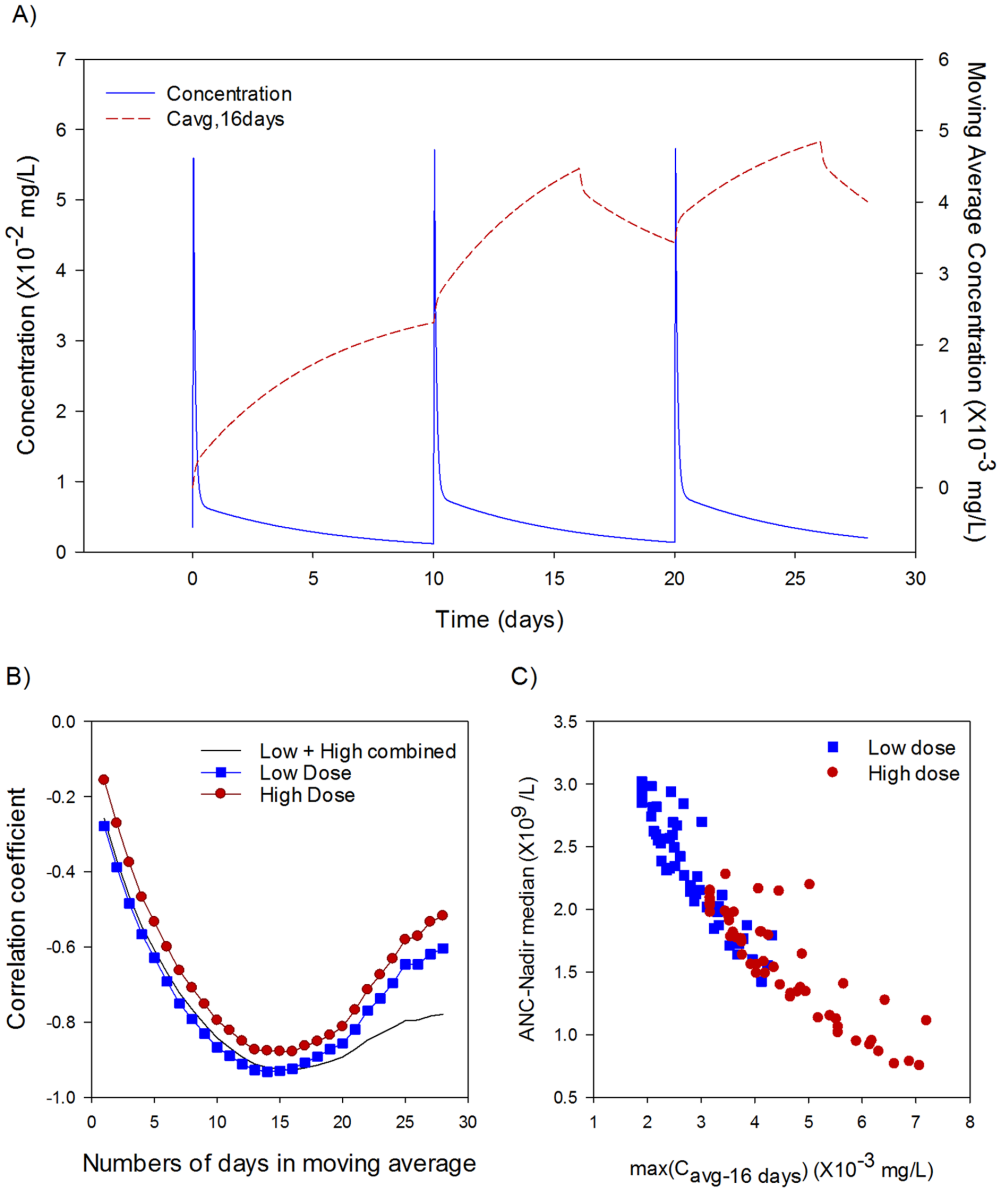
Moving average of PK describes neutropenia. The maximum of the moving average concentration over the dosing interval was examined for its ability to predict the simulated median ANC nadir. (A) Example of the 16-day moving average calculated from concentration profile from 1on-9off dosing schedule. (B) The correlation between maximal moving average concentration and ANC nadir was calculated for sliding windows of 1 day to 28 days for low dose (blue squares), high dose (red circles), and combination (black line). The maximum ability to predict neutropenia for the combined low and high total doses occurs when the moving average is calculated over 16 days. (C) The maximum of the moving average concentration, max(C_avg,16day_), accounts for most of the variability in median ANC nadir across total dose and schedule (R^2^ = 0.86, *p*<0.01).

Based on the prediction that frequent low doses of docetaxel would induce less neutropenia than infrequent large doses, we hypothesized the existence of a PK parameter for each schedule that would correlate directly with the simulated ANC nadir. To this end, we first examined two common PK parameters, C_max_ and total cycle AUC, for correlation with the median ANC nadir. The schedule-dependent C_max_ correlates poorly with the median ANC nadir (R^2^ = 0.10, *p*<0.05; Figure 3a). The total cycle AUC was also tested to see if it could explain the schedule-to-schedule differences in ANC nadir. The very low correlation between total cycle AUC and the median ANC nadir (R^2^ = <0.01, *p*>0.75) suggests that AUC alone is not fully accounting for the neutropenia effect. It is not surprising that AUC is not correlated, since total cycle AUC should be constant for linear PK and a fixed total dose.

Since total cycle AUC and C_max_ are not strongly associated with neutropenia at a fixed totally cycle dose, we sought another PK parameter that would be a better predictor of neutropenia. We examined the model under simplified linear conditions and found that the circulating neutrophil counts over time could be approximated analytically by a weighted moving average of the drug concentration over time (see Methods S1). We therefore tested a PK parameter (c_avg,ndays_) based on the moving average concentration, which only considers concentrations over a set window of time (n_days_). At each point in time, the moving average concentration is simply the average concentration over a set preceding interval of time, and is related to AUC over n_days_. The moving average is therefore capturing drug exposure occurring in the recent past (set by n_days_) but not exposures in the very distant past.

To assess the ability of this parameter to predict the neutropenia nadir, however, we must determine the duration over which the moving average is calculated, n_days_. To accomplish this, we assessed the correlation between median neutropenia nadir (maximal neutropenia) and max(c_avg,ndays_) over all schedules. The time-interval over which the moving average was calculated, n_days_, was varied from 1 day to 28 days. The correlation is plotted in Figure 3b, showing that the parameter correlates best with a moving average window set to 16 days. In Figure 3c the median ANC nadir is compared with max(c_avg,16days_) for all tested schedules, and is found to be a good predictor of the incidence of grade 4 neutropenia (R^2^ = 0.86, *p*<0.01). As a further test, we also examined the ability of max(c_avg,16days_) to predict the degree of neutropenia within the high and low total dose levels separately, and this again shows a strong correlation (R^2^ = 0.77 (*p*<0.01) and R^2^ = 0.85 (*p*<0.01) for high and low total dose respectively). This strong correlation suggests that the moving average is a good predictor of the neutropenia nadir.

This result suggests that the neutrophil system has a 'memory' of roughly two weeks in duration (10–20 days), meaning that this is roughly the amount of time needed for the stem cell compartment to have recovered sufficiently in response to a single bolus drug exposure. This parameter provides a quick and easy principle for direct ranking of different dosing schedules based on the expected neutropenia effect. For example, schedules which have the greatest maximal exposure over a two week period will be expected to produce the most severe neutropenia nadir.

### Generalization of findings to other drugs

**Figure 4:**
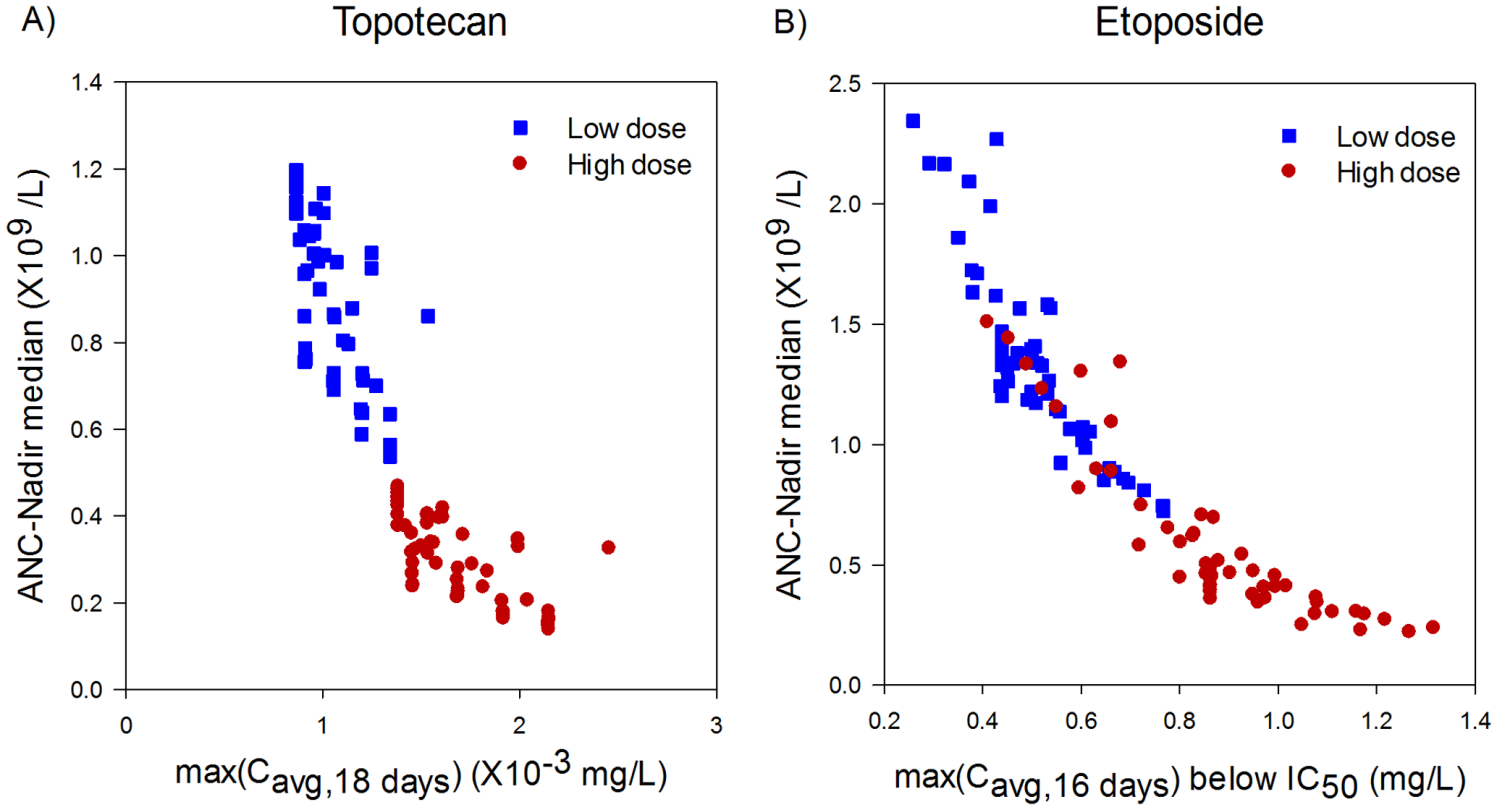
Consistency of moving average of PK in describing severity of neutropenia across drugs. The maximum of the moving average concentration over the dosing interval was examined for its consistency to predict neutropenia across different drugs. Topotecan induced neutropenia was simulated using linear drug effect model while in case of Etoposide, simulation was carried out using nonlinear drug effect (E_max_) model. (A) The maximum of moving average concentration over 18 days, max(C_avg,18day_), turns out to be a good predictor of Topotecan induced median ANC nadir(R^2^ = 0.79, *p*<0.01). While in case of Etoposide the maximum of moving average concentration below threshold over 16 days i.e IC_50_, max(C_avg,16day_<IC_50_), shows maximum ability to predict Etoposide induced median ANC nadir across total dose and schedules(R^2^ = 0.84, *p*<0.01).

Having identified a PK parameter, max(c_avg,16days_), that highly correlates with the severity of neutropenia induced by docetaxel, we then sought to demonstrate that this parameter was an intrinsic property of the neutrophil system and not specific to docetaxel. The analysis was therefore extended to include both etoposide and topotecan (Figures S1, S2, and S3). To test if the moving average concentration, max(c_avg,16days_), is a property of the neutrophil system alone, we tested if this parameter was a good predictor independent of the drug inducing the neutropenia. For topotecan, which has a linear drug-effect model [26], [34], the moving average of concentration is strongly predictive for ANC nadir (R^2^ = 0.79, *p*<0.01; Figure 4a and Figure S1). On the other hand, compared to clinically relevant plasma exposures, etoposide was found to have a highly nonlinear relationship between drug concentration and effect on the stem cell compartment [26], [34]. To account for the nonlinearity, we therefore used the moving average of concentration below IC_50_ of the drug (see methods) and the moving average of this parameter again is strongly predictive (R^2^ = 0.84, *p*<0.01; Figure 4b and Figure S2).

### In vivo ANC analysis

**Figure 5:**
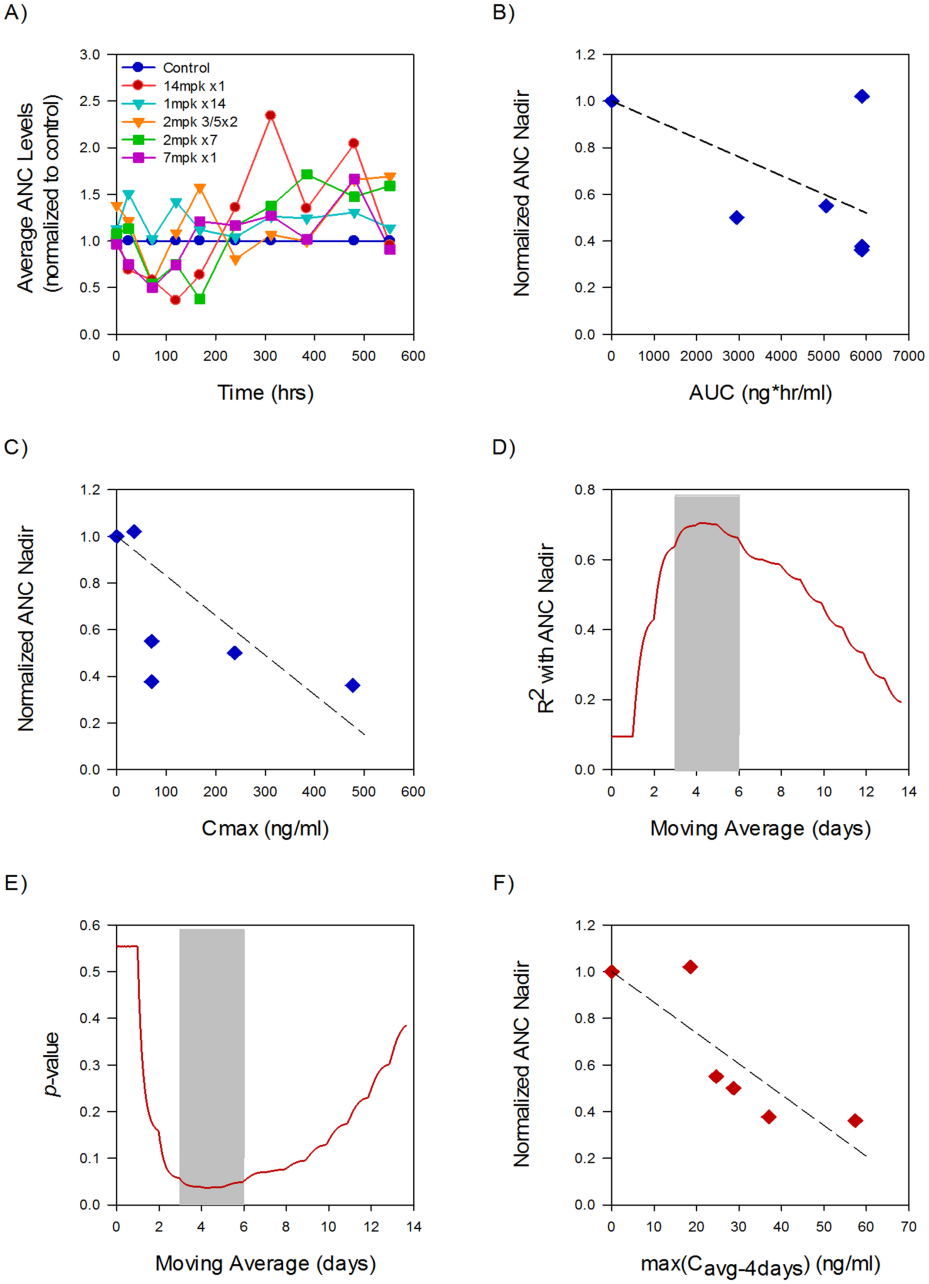
Moving average PK describes rat neutrophil counts. Rat neutrophil counts were measured response to the investigational PLK inhibitor TAK-960 and the relationship between plasma PK and neutrophil nadir was assessed. A) Absolute Neutrophil Count (ANC) normalized to control group plotted over the course of 21 days on a variety of dosing regimens. B) Normalized ANC nadir plotted versus total cycle AUC for each schedule tested. C) Normalized ANC nadir plotted versus the C_max_ for each schedule tested. D) R^2^ plotted for C_avg,ndays_ for n-days from 1 to 14 days. E) The corresponding p-values of the C_avg,ndays_ correlation showing correlation. F) Normalized ANC nadir is highly correlated with max(C_avg,4days_) with R^2^ = 0.70 (*p*<0.05), a better correlation than either C_max_ or AUC.

We next sought to determine if the PK parameter determined from the model was appropriate for analyzing *in vivo* data. To this end, we measured ANC levels in rats after administration of TAK-960, an investigational inhibitor of Polo-Like Kinase (PLK), a target known to induce neutropenia [43]. A variety of schedules and dose levels were tested to study the correlation between ANC nadir and PK in this system, each giving a different C_max_ and AUC which were calculated from a rat PK model (Figure S4). The time-course of circulating neutrophils was then assessed and normalized to control (Figure 5a). From this, the PK-ANC correlation was tested for AUC (Figure 5b) and C_max_ (Figure 5c), and shows a weak correlation for both (R^2^ = 0.18 (*p* = 0.40) and R^2^ = 0.09 (*p* = 0.55)), respectively. Since rat neutrophil development has a different timescale than in human [36] we again adjusted the n-days over which we tested the maximal moving average correlation with neutrophil nadir. For n-day values between 3 and 6, the correlation is strong (R^2^ = 0.70) and statistically significant (*p*<0.05) (Figure 5d-5e). The ∼3- fold difference between the nadir-predictive moving average between human (16 days) and rat (3–6 days) can be explained by the differences in the mean transit time in the human and rat neutropenia models [26], [36].

## Discussion

Identifying the PK parameter related to a pharmacological effect is important for schedule optimization, as well as for assessing dose-response relationships in the clinic. Often, when looking at clinical data, one has only one or two schedules to compare, which can make it difficult to decouple the effect of PK parameters which are often highly correlated between tested schedules. In this work we have attempted to bridge this gap by using a hybrid approach based on systems pharmacology and dose-response analysis. First we identified a potential PK driver by probing and mathematically analyzing the dynamic models built from clinical time-course of pharmacodynamic endpoints, and then validated the PK parameter retrospectively from experimental *in vivo* data. The result is a PK parameter that can be prospectively applied to analyze and select between alternative dosing schedules.

We found that the relationship between neutropenia nadir and schedule was not well explained by either total cycle AUC or C_max_. For AUC, this parameter is often more closely related to the total cycle dose and number of dosing cycles, than it is to the schedule of administration. For example, a patient undergoing treatment for two cycles would have twice the total cycle AUC of a patient undergoing a single cycle of treatment, but would not necessarily be expected to endure twice the nadir depth as a patient exposed to a single cycle. Similarly, C_max_ can often correlate more closely with the size of the dose more than with the schedule of administration. With regard to neutropenia, a second dose given before the stem cell compartment has fully recovered will be expected to induce a more severe nadir than a second dose given after stem cell compartment has fully recovered to pretreatment levels.

On the other hand neutrophil counts will eventually return to baseline sometime after drug has been cleared from the system, so at any point in time the system response is likely determined by drug exposures experienced in the recent past (e.g. a few days ago) but not in the distant past (e.g. over a year ago). Neutropenia can therefore be thought of as a transient response, which 'remembers' events up to some point in the past but 'forgets' events that occurred prior to that. In this context, it is not surprising, then that C_max_ and AUC did not faithfully capture the severity of neutropenia induced by various schedules. C_max_, for example, is a parameter with an infinitely short 'memory' and only represents the maximal instantaneous concentration (see Equation 2). On the other hand total AUC has an infinitely long 'memory', treating drug exposure from the recent past equally as strongly as drug exposures that occurred in the very distant past (see Equation 3).

Instead, based on an analytical analysis of the Friberg model of the transient neutrophil response to drug, we introduced a PK parameter based on the moving average concentration which captures the timescales inherent in the development and recovery of neutropenia. This parameter is related to the AUC that the system sees over a specific time period, rather than over the entire treatment duration, and it provides a quick and easy method for comparing dosing schedules. For example, it suggests that schedules with a lower total exposure given over a two week interval tend to produce less severe neutropenia nadirs and therefore lower probability of neutropenia incidence.

This model-derived insight differs from the conventional wisdom on the dosing schedule development for agents that induce neutropenia, where the goal is typically to space doses apart widely to provide enough time for the bone marrow to recover. Our work demonstrates that in many cases lower dose levels with more frequent administration of drug induced less neutropenia than larger infrequent doses, which is consistent with the published literature on taxanes [8]–[12], [25]. This finding has both a clinical and biological relevance for the future development of agents that induce neutropenia. Findings such as this also point to the value of a simplified PK parameter (such as the moving average) in decision-making, as it provides a simple way to assess clinical exposure-response data for homeostatic processes like production of circulating neutrophils.

When evaluating any model, it is especially important to keep in mind the assumptions that went into constructing it. One assumption that went into this analysis, is that inter-patient variability is constant across schedules being tested. For example, interpretation of results from clinical trials with different formulations [44] can be complicated by differences in magnitude and inter-individual variability of the bioavailability between the two formulations [45]. Similarly, it is worth noting the shape of the antiproliferative concentration-effect relationship for a compound. Based on previous model fitting exercises which assessed the best model fit for neutropenia, docetaxel and etoposide were both simulated using an *E_max_* model for concentration effect rather than the simple linear concentration-effect relationship that was found to fit best for topotecan. At the low, clinically relevant, concentrations, docetaxel effectively had a nearly linear relationship since concentrations spent little time above IC_90_. To demonstrate how to correct for highly nonlinear compounds, we used the example of etoposide which had a low IC_50_ relative to plasma concentration, resulting in a different rank order of schedules for the compound (Figure S5). Here instead, we examined average concentration below IC_50_, and found the moving average was still able to accurately capture the variability between schedules.

Clinical utility is a combination of safety and efficacy, and the goal of this study was to identify the predominant schedule effects driving neutropenia, a common safety endpoint. In many cases, total cycle exposure is likely to be an important determinant of efficacy. For example, dose fractionation of taxanes in the clinic have led to sustained efficacy (8, 10), and preclinical work often shows linear or saturating efficacy with increased concentration (46). In these scenarios, directly comparing schedules with equivalent total exposure as done in this study is appropriate, as schedules that maintain total exposure would not be expected to reduce efficacy even when resulting in a lower C_max_. Even for compounds when total cycle exposure is not the key determinant, the results we present with respect to identification of PK driver of neutropenia can still be used in conjunction with a similar PK determinant of efficacy to identify an optimal schedule. These results provide guidance for prioritization of schedules from a physiological perspective, and would therefore need to be verified directly in the clinic. Any decisions of clinical utility of an optimized schedule would need to then be considered from a cost-benefit analysis before implementation.

The moving average concentration has the inherent ability to capture a wide variety of timescales and can elicit properties of either C_max_ or AUC depending on the timescale over which the average is calculated. For example when the moving average window is very short compared to the PK of the compound (e.g. n_days_ on the order of hours) the max(c_avg,ndays_) closely reflects C_max_ respond acutely to instantaneous concentration of drug. On the other hand, for a longer moving average window (e.g. n_days_ on the order of weeks or months), max(c_avg,ndays_) more closely correlates with a total cycle AUC, and would therefore reflect the entire history of drug exposure.

It is likely that the concept of moving average concentration could be applicable to other types of toxicity beyond neutropenia. As with neutropenia, the moving average timescale could be taken to represent the inherent time over which any toxic endpoint is induced and transiently recovers. The moving average is a feature of the memory of such a system, and would therefore apply to cases where homeostatic mechanisms bring the system back to baseline sometime after pharmacological intervention is removed. Once the timescale (n_days_) for a particular toxicity is understood, it can become much easier to develop optimal dosing schedules to avoid inducing toxic events. We thus expect that the moving average concentration concept introduced here may have broad applicability as a PK parameter in analysis of many types of pharmacological activities, regardless of the timescale over which they develop and dissipate.

## Methods

### Ethical Statement

This study was performed in strict accordance with the recommendations in the Guide for the Care and Use of Laboratory Animals to ensure good science and animal care and welfare through compliance with internal policies as well as external regulatory agencies. The protocol was reviewed and approved for compliance with these regulations prior to study initiation by Millennium's Institutional Animal Care and Use Committee IACUC Committee (Protocol Number: 08-009, Study # DSD-01321). All animal were sacrificed at end of experiment using surgery using pentobarbital (iv) with accepted American Veterinary Medical Association (AVMA) guidelines, and every effort was made to minimize suffering.

### Neutropenia Model

The semi-mechanistic PK-PD model described by Friberg et al. [26], has been used in the current study and is shown in Figure 1a. The population PK-PD simulation was carried out using parameters in references [26], [34] with inter-individual variability incorporated on Circ0, MTT and drug effect parameters Slope or EC_50_. Here non-linear drug effect (E_max_) model was used to simulated ANC profiles upon docetaxel and etoposide administration, while topotecan induced myelosuppression was simulated using linear drug effect model (Table 2). Plasma concentration time profiles for all three drugs were simulated using published clinical PK models [27], [30], [46]. Using the PK-PD model, overall effect of drug fractionation on neutrophil counts was simulated for various schedules by keeping the total dose constant. All simulations were done for 84 days (corresponding to 28 days/cycle ×3 dosing cycles). The pharmacokinetics (PK) of topotecan, etoposide and docetaxel were simulated using typical population PK model parameters (Table S1). PK profiles were simulated for a wide range of schedules maintaining clinical recommended total doses (high dose and low dose, Table 1). Docetaxel was dosed as an IV bolus while topotecan and etoposide were dosed as short 30 minute infusions. Population PK-PD simulation was carried out using NONMEM Ver 7.1 for populations of 1000 virtual patients. Population simulations were analyzed with MATLAB (Mathworks, Natick, MA) and ANC nadir (median), ANC Nadir (90^th^ percentile), probability of grade-3 neutropenia (ANC less than 1×10^9^ neutrophils/liter) and probability of grade-4 neutropenia (ANC less than 0.5×10^9^ neutrophils/liter) were estimated. Probability of grade-4 neutropenia for more than seven continuous days was also calculated for each schedule from population simulations (Figure S6). The AUC of plasma concentration time profile for all drugs was calculated by noncompartmental analysis using the linear trapezoidal rule. The Pearson correlation coefficient was calculated in excel to measure correlation between two arrays of interest. Linear regression analysis was performed using graphpad PRISM (GraphPad Software, Inc., CA).

**Table 2:** Pharmacodynamic model parameters used in the simulation of ANC kinetics.

DRUG	Circ0 (X10^9/^L)	MTT (hours)	Drug Effect (E_drug_)	γ	Reference
Docetaxel	5.05	88.7	Emax Model	0.161	Friberg et al. 2006
			I_max_ = 83.9		
			IC_50_ = 7.17 uM		
Topotecan	5.02	137	Linear Model	0.101	Kloft et al. 2006
			Slope = 60.8 uM^−1^		
Etoposide	5.45	135	Emax Model	0.174	Friberg et al. 2006
			I_max_ = 1.57		
			IC_50_ = 5.2 uM		

### Rat PK and ANC measurements

Single dose of TAK-960 was administered via oral gavage to male Sprague Dawley rats (n = 3), 8–10 weeks of age, at 7 mg/kg and 14 mg/kg to estimate the PK profile. Blood samples were collected at 0, 0.5, 1, 2, 4, 8 and 24 hours. All blood samples were centrifuged to obtain plasma and freeze at ≤−70°C till analysis. A one-compartmental extravascular PK model adequately described the concentration-time profile of TAK-960 in rats (Figure S4). The PK model fitting and PK simulations were performed using Phoenix (Pharsight).

A repeat-dose hematological toxicity study of TAK-960 administered via oral gavage was performed on male Sprague Dawley rats. Six study groups with six rats per group were given the following PO schedules: vehicle (0.5% methylcellulose 1500 cps), 1 mg/kg QD days 1–14, 2 mg/kg QD days 1–7, 2 mg/kg 3on/4off 2 cycles, 7 mg/kg single dose and 14 mg/kg single dose. C_max_ and AUC estimates from the PK model for each schedule are provided in the Table S2. Blood samples were collected periodically to estimate the absolute neutrophil count.

*Comparison of PK Parameters: Maximal Moving Average, Total Cycle AUC, and Cmax:*

The moving average concentration is defined over a time interval, n_days_, using the formula in Eq. 1 and was calculated from simulated concentration profiles. Pretreatment concentrations (t<0) are assumed to be zero.
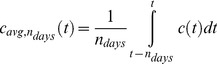
(Eq.1)


The parameter max(c_avg,ndays_) can be thought of as blending properties of both C_max_ and AUC. For example, in the limit where n_days_ is very small compared to the half-life of the molecule, max(c_avg,ndays_) is roughly equivalent to c_max_ on any given schedule(Eq. 2). 
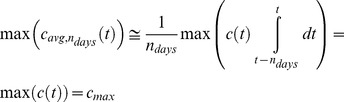
(Eq.2)


On the other hand, when n_days_ is very large compared to the duration of treatment, the parameter max(c_avg,ndays_) will be more related to the total cycle AUC for each schedule (Eq. 3).
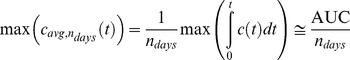
(Eq.3)


For Etoposide, the C_max_ was in some cases much larger than IC_50_. To account for this higher degree of nonlinearity we used the moving average of the concentration below IC_50_ defined by:
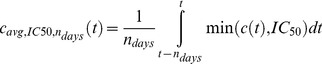
(Eq.4)


## Supporting Information

Figure S1 **Effect of schedule on topotecan induced neutropenia.** Population estimation of median ANC nadir and probability of grade 4 neutropenia (nadir <0.5×10^9^/L) from PK-PD simulation for topotecan shows similar patterns across schedules. The top row (A-B) represents median ANC nadir and probability of grade 4 neutropenia for all different schedules at low total dose while the bottom row (C-D) represents same for high total dose. Frequent dosing (schedules where ‘days-on’ is close to or equal the dosing period, such as 7on/0off) is associated with less probability of grade 4 neutropenia.
(TIF)

Figure S2 **Effect of schedule on etoposide induced neutropenia.** Population simulation of etoposide induced neutropenia was carried our using non-linear drug effect (Emax) model. The top row (A-B) represents median ANC nadir and probability of grade 4 neutropenia for all different schedules at low total dose while the bottom row (C-D) represents same for high total dose. Here intermittent dosing is associated with low probability of grade 4 neutropenia and higher ANC nadir.
(TIF)

Figure S3 **C_max_ and AUC are weak predictor of severity of neutropenia.** Common PK parameters were tested for its correlation with median ANC nadir and probability of grade-4 neutropenia across variety of schedules and dose for topotecan and etoposide. (A-C) C_max_ plotted against median ANC nadir for each of the schedules tested and shows weak correlation of −0.29 (R^2^ = 0.08, *p* = 0.002) and −0.31 (R^2^ = 0.09, *p* = 0.001) for topotecan and etoposide respectively. (B-D) Total cycle AUC over all schedules shows overall good correlation (CC = −0.82 (R^2^ = 0.67) and −0.72 (R^2^ = 0.50, *p*<0.01) for topotecan and etoposide respectively) with median ANC nadir, but the correlation is mainly driven by the differences in total dose as it loses ability to predict neutropenia at a fixed total dose level (R^2^<0.05 at low and high total dose for both drugs).
(TIF)

Figure S4 **Rat plasma PK model fits and model parameters.** One compartment pharmacokinetic model adequately describes TAK-960 plasma disposition/elimination after IV administration. Model fits and model parameters are shown in figure S4.
(TIF)

Figure S5 **Moving average concentration predicts neutropenia across drugs.** Maximal moving average concentration of n days, max(c_avg,n-days_), was found to predict degree of neutropenia precisely except highly nonlinear drug effect model of etoposide. In case of etoposide maximum moving concentration below threshold i.e. IC_50_ for 16 days, max(C_avg,16days_<IC_50_) turn out to be a good predictor of median ANC nadir and probability of grade-4 neutropenia. The median ANC nadir (top row) and probability of grade-4 neutropenia (bottom row) was plotted against moving average concentration, max(c_avg,n-days_).
(TIF)

Figure S6 **Schedule-dependence of grade-4 neutropenia for greater than seven days.** Probability of an individual presenting with grade-4 neutropenia continuously for seven days or more derived from PK-PD simulation for all three drugs is shown. The upper row (A-C) represents probability of grade-4 neutropenia continuously for seven days over all schedules tested at low total dose and lower row (D-F) represents same for high total dose of each drug. This parameter tends to favor dosing schedules with some dose holiday (e.g. 7on/7off). However, as with probability of a grade-4 event, schedules such as 1on/27off are suboptimal under this analysis.
(TIF)

Table S1 **Pharmacokinetic model parameters used in simulation of concentration time profiles.**
(DOCX)

Table S2 **PK parameters, AUC and C_max_ on each of the schedules tested.**
(DOCX)

Methods S1 (DOCX)
