# Supplementary figures and images for: Dose Schedule Optimization and the Pharmacokinetic Driver of Neutropenia
Source: PLoS One. 2014 Oct 31;9(10):e109892. doi: 10.1371/journal.pone.0109892 (PMC4215876; doi:10.1371/journal.pone.0109892)

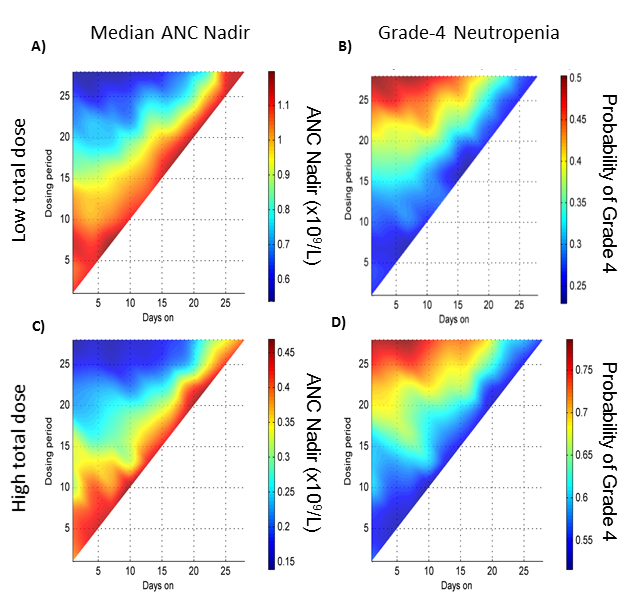

Supplement: Figure S1 — Effect of schedule on topotecan induced neutropenia. Population estimation of median ANC nadir and probability of grade 4 neutropenia (nadir <0.5×10^9^/L) from PK-PD simulation for topotecan shows similar patterns across schedules. The top row (A-B) represents median ANC nadir and probability of grade 4 neutropenia for all different schedules at low total dose while the bottom row (C-D) represents same for high total dose. Frequent dosing (schedules where ‘days-on’ is close to or equal the dosing period, such as 7on/0off) is associated with less probability of grade 4 neutropenia. (TIF) [file pone.0109892.s001.tif]

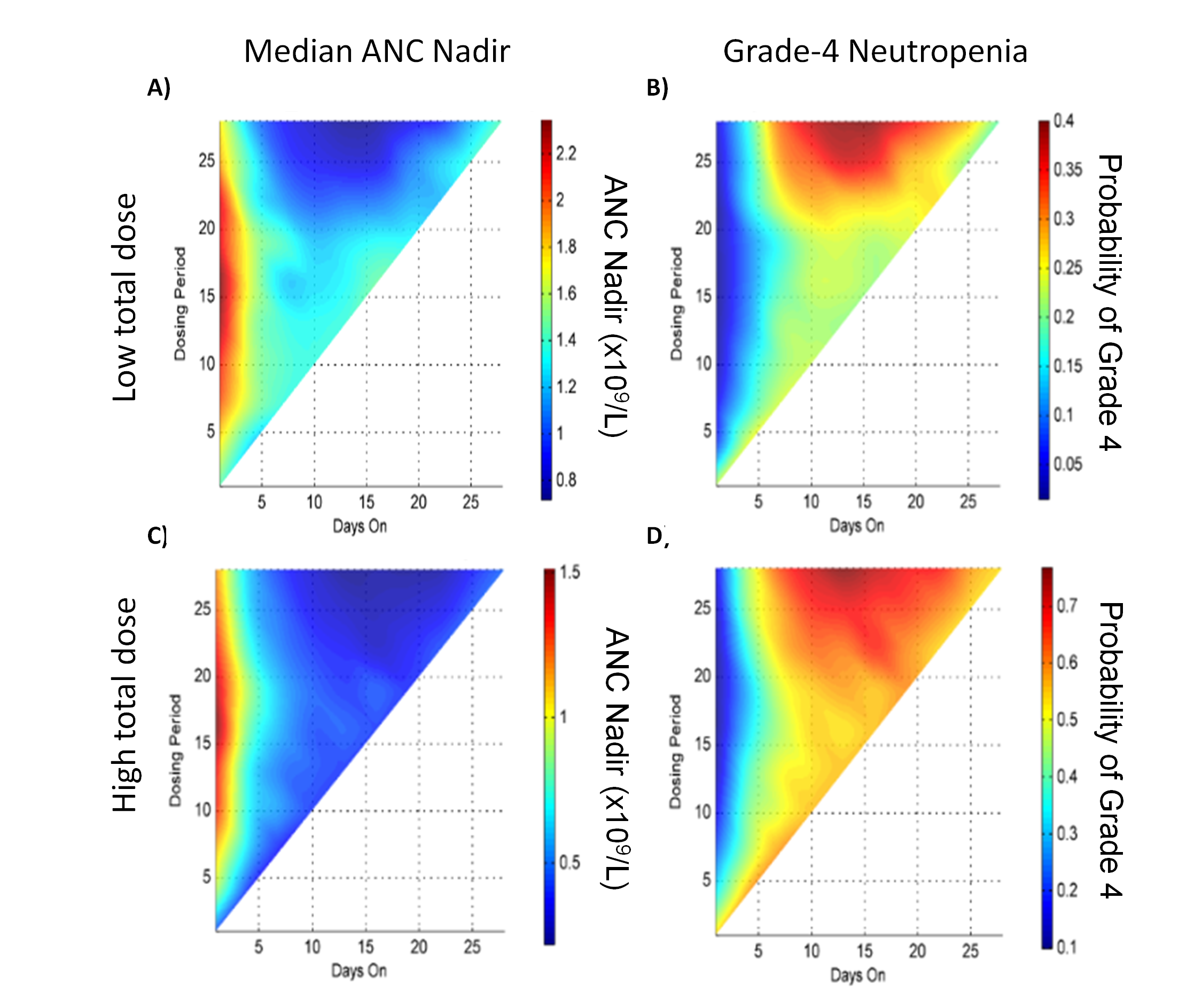

Supplement: Figure S2 — Effect of schedule on etoposide induced neutropenia. Population simulation of etoposide induced neutropenia was carried our using non-linear drug effect (Emax) model. The top row (A-B) represents median ANC nadir and probability of grade 4 neutropenia for all different schedules at low total dose while the bottom row (C-D) represents same for high total dose. Here intermittent dosing is associated with low probability of grade 4 neutropenia and higher ANC nadir. (TIF) [file pone.0109892.s002.tif]

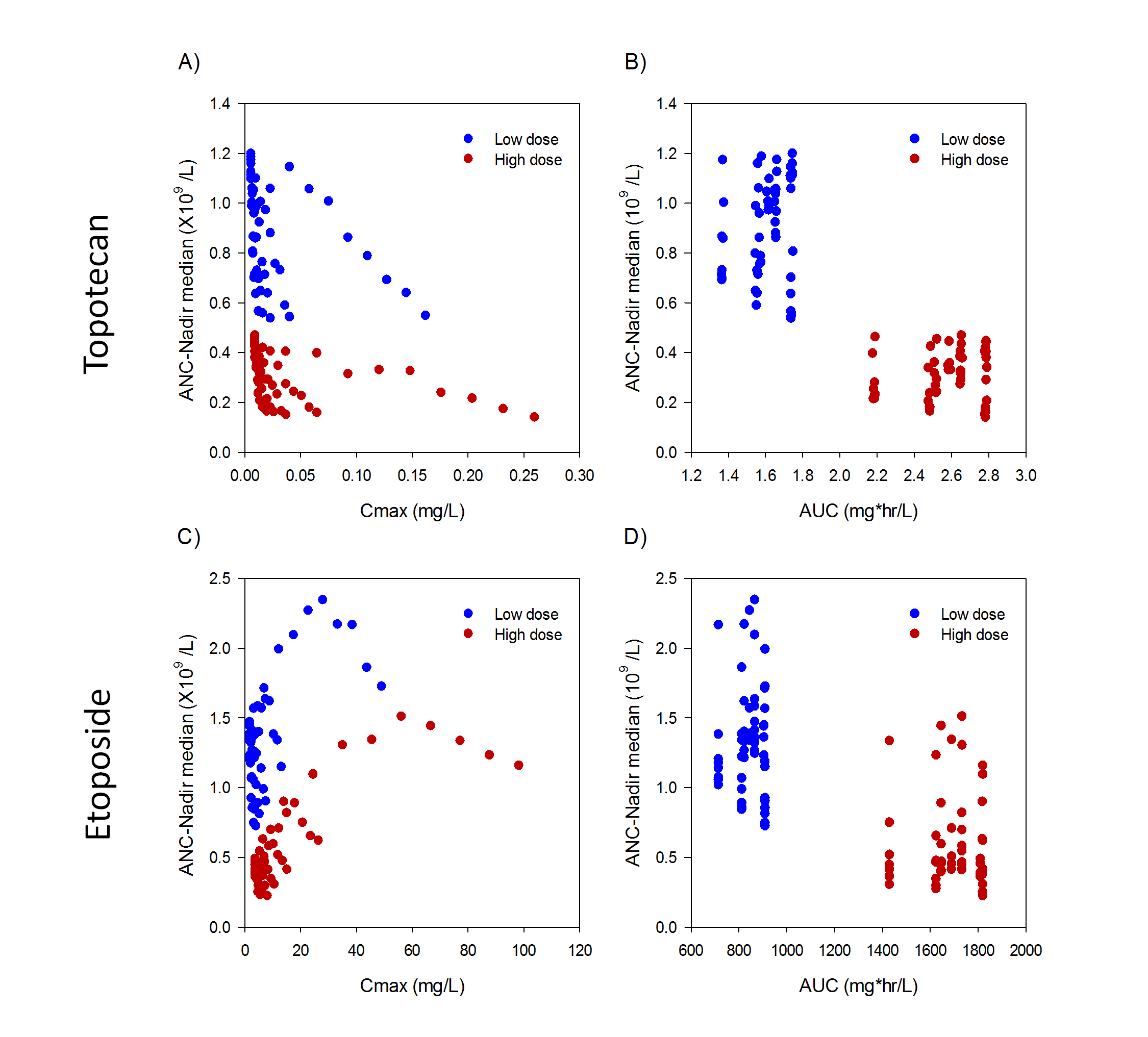

Supplement: Figure S3 — Cmax and AUC are weak predictor of severity of neutropenia. Common PK parameters were tested for its correlation with median ANC nadir and probability of grade-4 neutropenia across variety of schedules and dose for topotecan and etoposide. (A-C) Cmax plotted against median ANC nadir for each of the schedules tested and shows weak correlation of −0.29 (R2 = 0.08, p = 0.002) and −0.31 (R2 = 0.09, p = 0.001) for topotecan and etoposide respectively. (B-D) Total cycle AUC over all schedules shows overall good correlation (CC = −0.82 (R2 = 0.67) and −0.72 (R2 = 0.50, p<0.01) for topotecan and etoposide respectively) with median ANC nadir, but the correlation is mainly driven by the differences in total dose as it loses ability to predict neutropenia at a fixed total dose level (R2<0.05 at low and high total dose for both drugs). (TIF) [file pone.0109892.s003.tif]

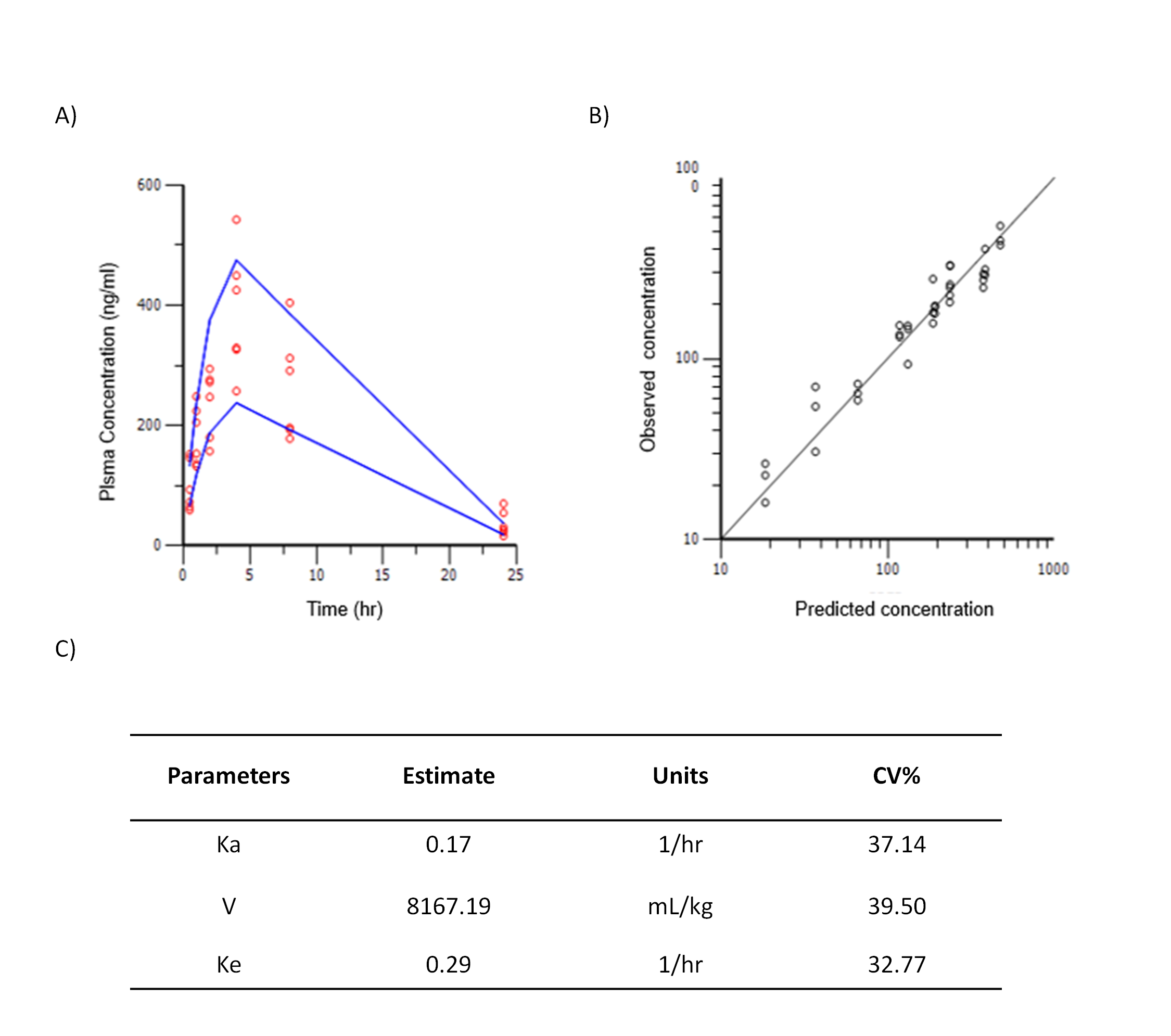

Supplement: Figure S4 — Rat plasma PK model fits and model parameters. One compartment pharmacokinetic model adequately describes TAK-960 plasma disposition/elimination after IV administration. Model fits and model parameters are shown in figure S4. (TIF) [file pone.0109892.s004.tif]

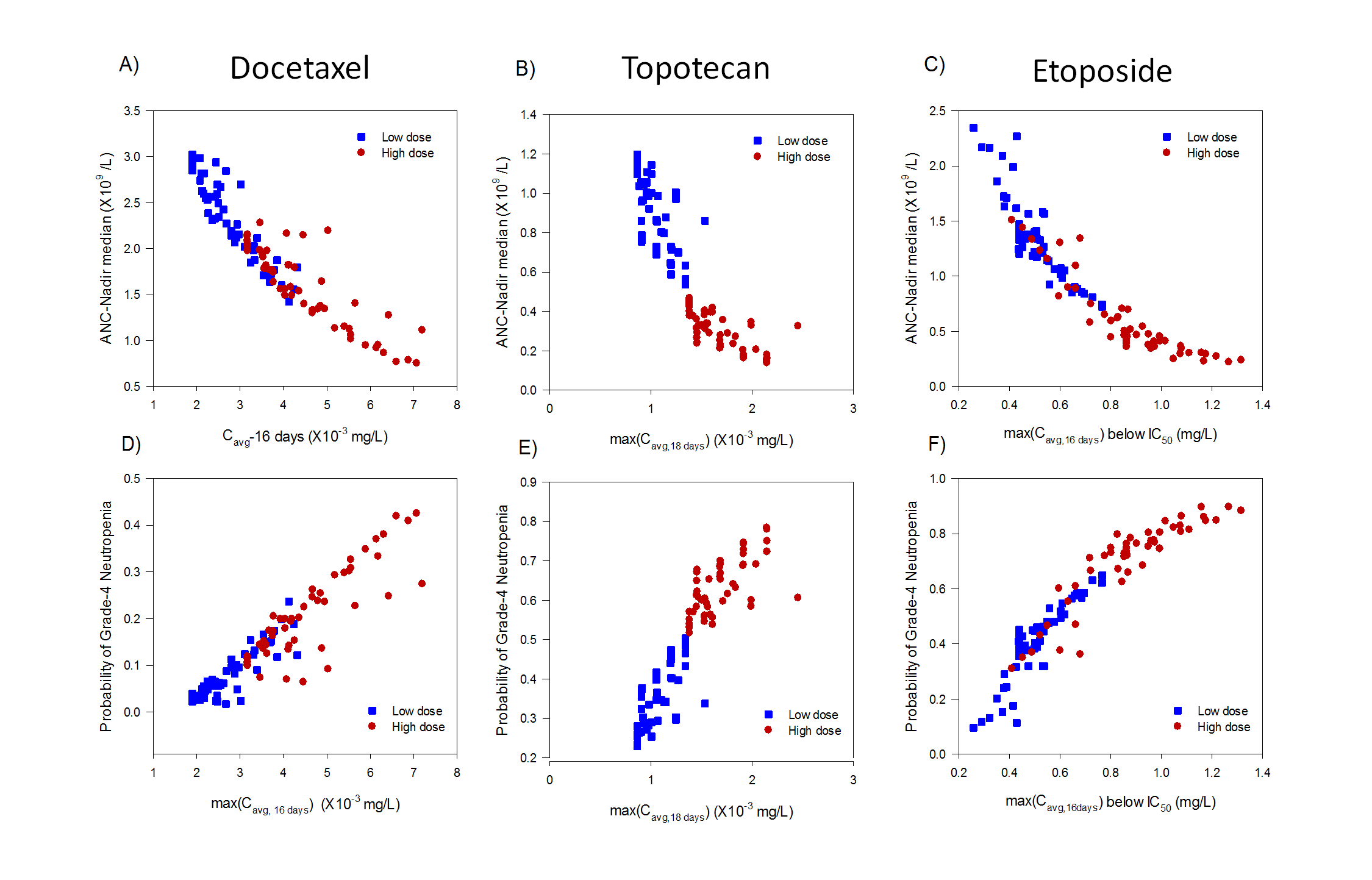

Supplement: Figure S5 — Moving average concentration predicts neutropenia across drugs. Maximal moving average concentration of n days, max(cavg,n-days), was found to predict degree of neutropenia precisely except highly nonlinear drug effect model of etoposide. In case of etoposide maximum moving concentration below threshold i.e. IC50 for 16 days, max(Cavg,16days<IC50) turn out to be a good predictor of median ANC nadir and probability of grade-4 neutropenia. The median ANC nadir (top row) and probability of grade-4 neutropenia (bottom row) was plotted against moving average concentration, max(cavg,n-days). (TIF) [file pone.0109892.s005.tif]

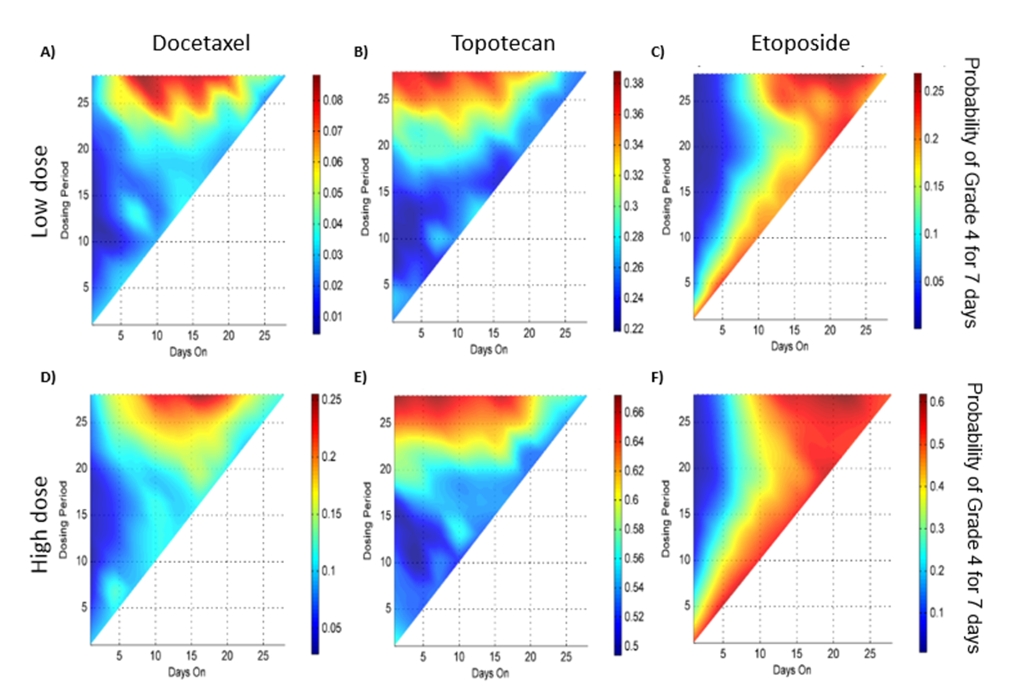

Supplement: Figure S6 — Schedule-dependence of grade-4 neutropenia for greater than seven days. Probability of an individual presenting with grade-4 neutropenia continuously for seven days or more derived from PK-PD simulation for all three drugs is shown. The upper row (A-C) represents probability of grade-4 neutropenia continuously for seven days over all schedules tested at low total dose and lower row (D-F) represents same for high total dose of each drug. This parameter tends to favor dosing schedules with some dose holiday (e.g. 7on/7off). However, as with probability of a grade-4 event, schedules such as 1on/27off are suboptimal under this analysis. (TIF) [file pone.0109892.s006.tif]
