# Supplementary material for: Dose Schedule Optimization and the Pharmacokinetic Driver of Neutropenia
Source: PLoS One. 2014 Oct 31;9(10):e109892. doi: 10.1371/journal.pone.0109892 (PMC4215876; doi:10.1371/journal.pone.0109892)
Supplement: Table S1 — Pharmacokinetic model parameters used in simulation of concentration time profiles. (DOCX) [file pone.0109892.s007.docx]

| **Compound** | **Pharmacokinetic model** | **Parameters** | | | | **Reference** |
| --- | --- | --- | --- | --- | --- | --- |
| Etoposide | Two-compartment model |  | CL | 1.14 | L/h | Toffoli, Corona et al. 2004 [[46](#_ENREF_46)] |
|  |  |  | V | 6 | L |  |
|  |  |  | K12 | 0.14 | 1/h |  |
|  |  |  | K21 | 0.06 | 1/h |  |
| Docetaxel | Three-compartment model |  | CL | 39.5 | L/h | Sandstrom, Lindman et al. 2005 [[27](#_ENREF_27)] |
|  |  |  | V | 11.1 | L |  |
|  |  |  | Q2 | 8.76 | L/h |  |
|  |  |  | V2 | 15.4 | L |  |
|  |  |  | Q3 | 31.3 | L/h |  |
|  |  |  | V3 | 2340 | L |  |
| Topotecan | Two-compartment model |  | CL | 25.48 | L/h | Leger, Loos et al. 2004 [[30](#_ENREF_30)] |
|  |  |  | V | 39.9 | L |  |
|  |  |  | Q | 49.9 | L/h |  |
|  |  |  | V2 | 44.5 | L |  |

**Table S1: Pharmacokinetic model parameters used in simulation of concentration time profiles**
