# Supplementary material for: Dose Schedule Optimization and the Pharmacokinetic Driver of Neutropenia
Source: PLoS One. 2014 Oct 31;9(10):e109892. doi: 10.1371/journal.pone.0109892 (PMC4215876; doi:10.1371/journal.pone.0109892)
Supplement: Table S2 — PK parameters, AUC and Cmax on each of the schedules tested. (DOCX) [file pone.0109892.s008.docx]

Table S2: PK parameters, AUC and C_max_ on each of the schedules tested

| **Schedule** | **AUC** | **C_max_** |
| --- | --- | --- |
| 1mpk x14 | 5897.935 | 35.3686 |
| 2mpk x7 | 5896.854 | 70.7269 |
| 2mpk 3/5x2 | 5054.698 | 70.7352 |
| 7mpk x1 | 2945.281 | 238.508 |
| 14mpk x1 | 5890.559 | 477.015 |
| Control | 0 | 0 |
