# Supplementary material for: Dose Schedule Optimization and the Pharmacokinetic Driver of Neutropenia
Source: PLoS One. 2014 Oct 31;9(10):e109892. doi: 10.1371/journal.pone.0109892 (PMC4215876; doi:10.1371/journal.pone.0109892)
Supplement: Methods S1 — (DOCX) [file pone.0109892.s009.docx]

**Supplemental method:**

To identify potential PK drivers for ANC nadir, we begin by analyzing a published model under simplifying conditions (26). Specifically we determine an analytical approximation to the model by linearizing the ordinary differential equation model and analyzing fluctuations around steady state in response to small perturbations. We first start with the general model equations from Friberg et al (26).

$\frac{dP}{dt}=K_{tr}P\left(1-E\right)\left(\frac{C_{0}}{C}\right)^{\gamma}- K_{tr}P$ (S1)

$\frac{dT_{1}}{dt}=K_{tr}\left(P-T_{1}\right)$ (S2)

$\frac{dT_{2}}{dt}=K_{tr}\left(T_{1}-T_{2}\right)$ (S3)

$\frac{dT_{3}}{dt}=K_{tr}\left(T_{2}-T_{3}\right)$ (S4)

$\frac{dC}{dt}=K_{tr}(T_{3}-C)$ (S5)

Where $\text{ }P,T_{1},T_{2},T_{3},C\text{ }$represents neutrophil counts in proliferation, transit1, transit2, transit3, and circulating neutrophils, respectively. $E\left(t\right)$represents the drug effect on the proliferating cell compartment. There are three transit compartments in the model (T_1_, T_2_ and T_3_) and transit rate across compartment is denoted as K_tr_. Mean transit time (MTT) is defined by the number of transit compartments and transit rate (K_tr_).

$MTT=\frac{Numbers of transit compartments+1}{Transit rate}=\frac{4}{K_{tr}}$ (S6)

The solution at steady state in the absence of drug ($E\left(t\right)=0)$ is:

$$C_{ss}=T_{3,SS}=T_{2,SS}=T_{1,SS}=P_{ss}=C_{0}$$

In the dynamic scenario, we approximate the transit compartments between $P\left(t\right)$ and $C\left(t\right)$ as a delayed differential equation using the Mean Transit Time ($MTT$) described in Friberg et al.

$C\left(t\right)\approx P\left(t-MTT\right)$ (S7)

Then defining fluctuations from $P_{ss} \text{as} \delta P\left(t\right)$ ;

$P\left(t\right)=P_{ss}+\delta P\left(t\right)$(S8)

Substituting these relationships leads to the following single delayed differential equation:

$\frac{d\left(\delta P\right)}{dt}\cong K_{tr}(P_{ss}+\delta P)\left(1-E\right)\left(\frac{C_{0}}{P_{ss}+\delta P\left(t-MTT\right)}\right)^{\gamma}- K_{tr}(P_{ss}+\delta P)$ (S9)

Under conditions of small perturbations,$\left|E\right|\ll1$, the system response will be commensurately small, $\left|\delta P\right|\ll1$, allowing the feedback relationship to be approximated as:

$\left(\frac{C_{0}}{P_{ss}+\delta P\left(t-MTT\right)}\right)^{\gamma}\cong1-\gamma\frac{\delta P}{C_{0}}$ (S10)

Substituting this relationship and expanding terms, we arrive at:

$\begin{matrix} \frac{1}{-K_{tr}}\frac{d\left(\delta P\right)}{dt}\cong C_{0}E+\gamma\delta P\left(t-MTT\right)- E\gamma\delta P\left(t-MTT\right)+E\delta P\left(t\right)+\frac{\gamma}{C_{0}}\delta P\left(t\right)\delta P\left(t-MTT\right) \\ - E\frac{\gamma}{C_{0}}\delta P\left(t\right)\delta P\left(t-MTT\right) \end{matrix}$ (S11)

Since we are interested in first order effects due to small perturbations (cases when $|E|\ll1$and therefore $|\delta P|\ll1$) we keep only first order terms from equation S11 , ignoring any second and third order terms.

$\frac{1}{-K_{tr}}\frac{d\left(\delta P\right)}{dt}\cong C_{0}E+\gamma\delta P\left(t-MTT\right)$ (S12)

Next, we concentrate on low frequency fluctuations over timescales longer than$MTT$, and approximate$\delta P\left(t-MTT\right)\cong\delta P-MTT\frac{d\left(\delta P\right)}{dt}$, yielding the equation:

$\left(\gamma MTT-\frac{1}{K_{tr}}\right)\frac{d\left(\delta P\right)}{dt}\cong C_{0}E+\gamma\left(\delta P\right)$ (S13)

Solving the differential equation and substituting the value for the delay$MTT=\frac{4}{K_{tr}}$ , yields the following relationship between drug effect and proliferating cells as a convolution of the impulse response function with the drug effect:

$\delta P\left(t\right)\cong\frac{K_{tr}C_{0}}{4\gamma-1}\int_{-\infty}^{t} E\left(\tau\right) e^{-\frac{\gamma K_{tr}}{1-4\gamma}\left(t-\tau\right)}d\tau$ (S14)

In the case of linear drug effect, $E\left(t\right)\cong slope*conc\left(t\right)$, the relationship becomes a weighted moving average of concentration:

$\delta P\left(t\right)\cong\frac{K_{tr}C_{0}Slope}{4\gamma-1}\int_{-\infty}^{t} Conc\left(\tau\right) e^{-\frac{\gamma K_{tr}}{1-4\gamma}\left(t-\tau\right)}d\tau$ (S15)

For purely illustrative purposes, one can note that the exponential kernel in the convolution is approximately 1 for (t-τ)<<[(1-4γ)/( γ*K_tr_)] and approximately zero for (t-τ)>>[(1-4γ)/( γ*K_tr_)]. If the exponential is therefore approximated by:

$$e^{-\frac{\gamma K_{tr}}{1-4\gamma}\left(t-\tau\right)}\sim\left\{\begin{matrix} 1 for \frac{\gamma K_{tr}}{1-4\gamma}\left(t-\tau\right)\leq1 \\ 0 for \frac{\gamma K_{tr}}{1-4\gamma}\left(t-\tau\right)>1 \end{matrix}\right.$$

Replacing above approximation in equation S15, we arrive at:

$\delta P\left(t\right)\cong\frac{K_{tr}C_{0}Slope}{4\gamma-1}\int_{t-\frac{1-4\gamma}{\gamma K_{tr}}}^{t} Conc\left(\tau\right)d\tau$ (S16)

In equation S16, $\delta P\left(t\right)$represents change in the proliferation compartment based on a moving average of the concentration simplified, which is related to the AUC over a time-frame corresponding to the memory of the system. Further, we can see from this equation, that the ANC nadir, $\text{min}\left(P\left(t\right)\right)$ or (max $\delta P\left(t\right))$ will be related to the maximum of the moving average of the concentration when $\gamma<0.25$. When considering this analysis we have to keep in mind the underlying assumptions that this solution will only apply to a highly linear case for small drug perturbations and will be more accurate for low frequency fluctuations. To understand how the system responds to larger perturbations, a further validation of moving average parameter is needed in experimental settings. This provides a basis for exploration of the maximal moving average concentration as a PK driver for neutrophil nadir under linear conditions. Further, this formula also suggest that under nonlinear conditions with saturating drug effect, a variation of the above calculation which accounts for the saturation as an area below IC50 should be examined.
